# Lipoid Proteinosis: Identification of a Novel Nonsense Mutation c.1246C>T:p.R416X in ECM1 gene from Bangladesh

**DOI:** 10.12669/pjms.39.4.7437

**Published:** 2023

**Authors:** Md. Azraf Hossain Khan, Md. Abu Reza, Ibrahim Md. Sharaf, Md. Jahangir Alam, Md. Mostafizur Rahman, Pampa Chandra, Kazi Selim Anwar, Md. Abdus Salam

**Affiliations:** 1 Prof. Md. Azraf Hossain Khan, DDV, MCPS Department of Dermatology & Venereology, Rajshahi Medical College Hospital, Rajshahi, Bangladesh; 2 Prof. Md. Abu Reza, PhD Molecular Biology and Protein Science Lab, Department of Genetic Engineering and Biotechnology, University of Rajshahi, Rajshahi, Bangladesh; 3 Ibrahim Md. Sharaf, DDV, MCPS;Medical Officer Department of Dermatology & Venereology, Rajshahi Medical College Hospital, Rajshahi, Bangladesh; 4 Md. Jahangir Alam, MS Molecular Biology and Protein Science Lab, Department of Genetic Engineering and Biotechnology, University of Rajshahi, Rajshahi, Bangladesh; 5 Md. Mostafizur Rahman, FCPS Assistant Professor Department of Dermatology & Venereology, Rajshahi Medical College Hospital, Rajshahi, Bangladesh; 6 Pampa Chandra, FCPS Medical Officer Department of Dermatology & Venereology, Rajshahi Medical College Hospital, Rajshahi, Bangladesh; 7 Kazi Selim Anwar, M.Phil. Head of Research Unit, Ad-din Research Unit (ARU), Ad-din Women’s Medical College, Bara Maghbazar, Dhaka, Bangladesh; 8 Prof. Md. Abdus Salam, PhD, FRCP (UK) Department of Basic Medical Sciences, Faculty of Medicine, International Islamic University Malaysia

**Keywords:** Lipoid proteinosis, Extracellular matrix protein 1 gene (ECM1), Nonsense mutation in exon-8, 1246(c.1246C>T), Hoarseness of voice, Bangladesh

## Abstract

Lipoid proteinosis is a rare multisystem genodermatosis inherited as autosomal recessive trait. We report a case of lipoid proteinosis in a 10-year-old boy born to first-degree consanguineous parents presented with marked hoarseness of voice, accelerated photoaging appearance, enlarged and erythematous tongue with restricted movement and widespread dermatoses. Biopsy of oral mucosa revealed Periodic acid-Schiff (PAS)-positive amorphous eosinophilic hyaline deposits. Mutational analysis revealed a homozygous nonsense mutation with C to T substitution at nucleotide position 1246(c.1246C>T) in exon-8 of the extracellular matrix protein 1 gene leading to a stop codon. Both the parents were unaffected heterozygous carriers. To our knowledge, this is the first case report of lipoid proteinosis with evidence of a novel nonsense genetic mutation from Bangladesh.

## INTRODUCTION

Lipoid proteinosis (LP) is a rare multisystem autosomal recessive genodermatosis, was first described in 1929 by Urbach and Wiethe. It occurs due to mutations in the extracellular matrix protein 1 gene (ECM1) on chromosome 1q21.2, comprising 10 exons. LP has essential attributes in blood physiology and skin anatomy leading to gross alteration in the microvasculature of dermis.^1^ Until to date, there are 66 different pathogenic ECM1 gene mutations have been described with both homozygous and compound heterozygous genotypes. Although various mutational types including frameshift, missense, nonsense, splice site, small and gross insertions and deletions affecting all 10 exons have been reported, overwhelming majority of mutations were in exon-6 and exon-7 and found to have missense or nonsense mutation.^2^,^3^

LP is characterized by generalized thickening and scaring of skin and mucosae and presents invariably by hoarseness of voice in infancy and subsequent appearance of yellowish, beaded papules and nodules in the skin result from abnormal deposition of hyaline material. Although rare the extracutaneous manifestations of LP may include epilepsy and neuropsychiatric abnormalities.^4^ As of to date, there are approximately 400 cases of LP reported worldwide with relatively higher incidence from South Africa and Turkey.^5^

To our knowledge this is the first case of LP from Bangladesh where a novel nonsense mutation in exon-8 of ECM1 gene has been explored to correlate with the phenotypic features. We believe our findings will add to the existing knowledge on LP, especially in the molecular characterization aspect of ECM1 gene and fill up the knowledge gap.

## CASE REPORT

**Fig.1:**
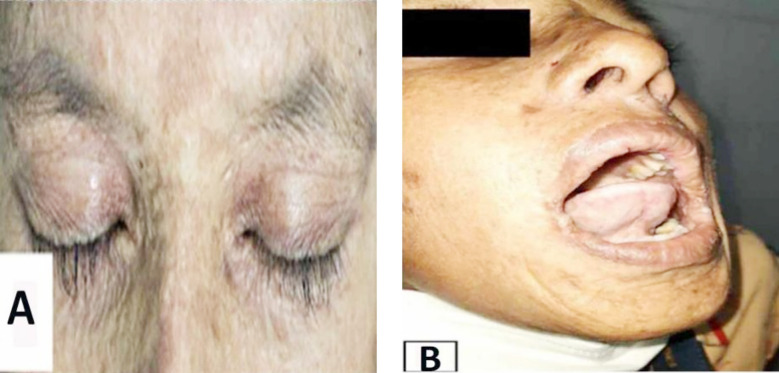
(A) Beaded papules along the eyelids; (B) Photoaging appearance with short frenulum of tongue.

A 10-year-old boy born to first-degree consanguineous parents was presented to the dermatology out patient department due to some skin problems noticed since early childhood. While talking to the patient, marked hoarseness of voice was noted and upon query, mother mentioned that he had a weak cry soon after birth that later developed gradually to hoarseness of voice and speech difficulty. She further added that while the boy was four-year-old, some blisters appeared on the face and tongue which ruptured spontaneously to cause painful swallowing of solid food. Since then he developed recurrent tongue sore, upper respiratory tract infection and blisters on different parts of the body which are tense, painful, non-pruritic and healed with scarring. Clinical examination revealed enlarged and erythematous tongue with restricted movement, eroded thickened skin lesions on the face, lips and other parts of the trunk with waxy yellowish papules, and atrophy in some areas (Fig.1). Generalized acneiform, atrophic lesions of various size and shape on the face, trunk and extremities led to accelerated signs of photoaging.

The boy has an unaffected 19-year-old sibling (sister), and both the parents are normal. His vision, hearing, urine, and bowel habits were normal. Respiratory, cardiovascular, and basic neurological and psychiatric examinations were normal and there is no history of convulsion. After obtaining institutional ethical clearance (Ref. No. RMC/ERC/2021/42; Date: 30-10-2021) and informed consent from the patient and assent from the parent, samples were collected for relevant investigations. Further, permission was sort for publishing photograph in case of scientific dissemination of knowledge.

**Fig.2:**
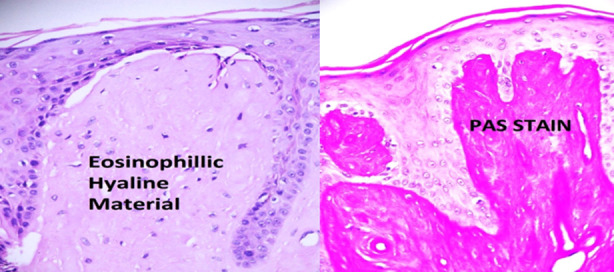
Histology of oral mucosa showing eosinophilic hyaline materials (Hematoxylin & Eosin) and positive Periodic acid-Schiff (PAS) reaction.

All investigations including CT scan & MRI of brain were normal. Fiber optic laryngoscopy (FOL) demonstrated thickened and edematous vocal cords, hypertrophied corniculate and arytenoid cartilage with fibrotic fold closing the laryngeal inlet. Biopsy of oral mucosa revealed amorphous eosinophilic deposits in dermis around the blood vessels, which were Periodic acid-Schiff (PAS)-positive and diastase resistant (Fig.2).

### Genetic analysis:

**Fig.3:**
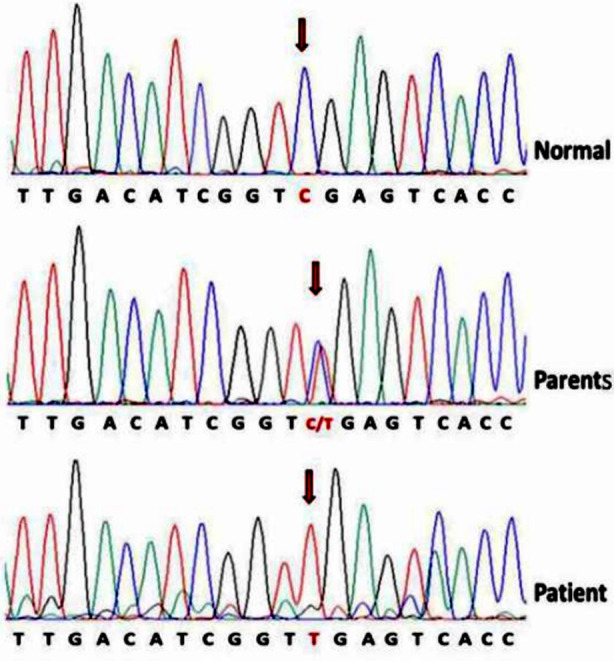
Chromatogram showing the homozygous nonsense mutation (c.C1246T:p.R416X) in patient and heterozygosity in parents in exon-8 of ECM1 gene.

Exome sequencing (Applied Biosystems Inc., USA) revealed a homozygous nonsense mutation with C to T substitution at nucleotide position 1246(c.1246C>T) in exon-8 of the ECM1 gene (Fig. 3), leading to a stop codon (p.R416X). Both the parents were heterozygous for the mutation and confirmed as unaffected heterozygous carriers.

## DISCUSSION

Lipoid proteinosis, also known as *hyalinosis cutis et mucosae* or Urbach-Wiethe disease, is predominantly characterized by abnormal deposition of hyaline material in the skin and mucosa causing a waxy and thick appearance of the skin.^6^ Yellowish infiltrated papules, nodules and verrucous hyperkeratosis appear on the skin especially areas that remain exposed to sun are more likely to be badly damaged and lead to photoageing appearance. Mucosal lesions always include vocal cord and laryngeal thickening, accompanied by variable tongue, palate, and lip infiltration resulting in hoarseness of voice. Pathogenesis may involve internal organs, but overall reliable clinical signs remain hoarseness of voice and thickened sublingual frenulum.

Although it is exceedingly rare, higher frequency has been observed in the Northern Cape province of South Africa, including Namaqualand and Turkey owing to a common founder effect. Cases are more frequent from countries where there is a culture of consanguineous marriage.^5^,^7^ Heterozygous carriers, estimated to be one in 400 of the general population, are asymptomatic.

Mutation in the ECM1 gene with a loss of function may result in an unusual pattern of keratinocyte maturation and differentiation, as well as disruption of dermal integrity and homeostasis.^8^ Clinically, LP has varied expressions and there are four ECM1 gene variants, which makes it even more difficult to correlate mutations with phenotypes. So far, more than 50 loss-of-function mutations, predominantly missense and nonsense mutations have been reported that can involve all 10 exons. Mutations most commonly affect exons-6 and 7, with exon-7 displays milder symptoms, while mutations in exon-6 cause a more severe phenotype.^2^,^3^ In our case, a nonsense mutation with C to T substitution at nucleotide position 1246(c.1246C>T) in exon-8 of the ECM1 gene has led to a stop codon (p.R416X), predicting premature termination of ECM1 protein with 415 amino acids. Similar mutation in exon-8 affecting codon for arginine at amino acid position 416 leading to a stop codon like ours has been previously reported (unpublished) in an Indian family but there are reports of other types of mutations affecting exon-8 in the literature.^4^,^9^ An Egyptian study in 2017 found five novel *ECM1* gene mutations in exon-1 (c.10-11insC), exon-6 (c.690-691delAG), exon-7 (c.734G>A), exon-8 (c.1286-1287delAA), and intron 9 (c.1393-1G>T) and all patients were phenotypically correlated with typical dermatoses and incidentally overwhelming majority were consanguineous.^10^ Although genetic mutation in ECM1 has been correlated as causal relation of LP, however, role of environmental factors, other genetic or epigenetic modifiers are yet to explore. Moreover, dermatological features need to be differentiated from metabolic disorders such as erythropoietic protoporphyria (EPP), amyloidosis, xanthomatosis, and papular mucinosis (scleromyxedema) through tissue interpretations and genetic analysis where possible.^11^

As far as the treatment of LP is concerned, there is no known effective treatment, however, it demands a multidisciplinary approach and needs to be tailored on individual basis depending on manifestations of disease.^12^ Further, consanguinity is an important risk factor for LP, genetic counselling and carrier screening of the parents should be a key task in prevention. Besides, patient education on avoidance of trauma, sun exposure, and use of sunscreen may aid in improving quality of life. The prognosis is variable, LP usually has a chronic but relatively benign course with a normal life expectancy.

## CONCLUSION

Lipoid proteinosis is a rare multisystem inherited disease. Physicians should be aware about various presentations of this condition to identify it early so that appropriate therapeutic measures can be taken. We consider this attempt as novel and believe that our findings will add on to the existing knowledge on lipoid proteinosis.

### Author`s Contribution:

**MAHK:** Conceptualization, clinical supervision, original draft and responsible for integrity of work.

**MAR:** Supervision of genetic analysis.

**IMS:** Clinical data collection, original draft.

**MJA:** Genetic analysis.

**MMR:** Follow-up of patient, literature review.

**PC:** Clinical data collection, literature review.

**KSA:** Revision and editing.

**MAS:** Review, editing and critical scientific inputs. All authors accepted the final version.
